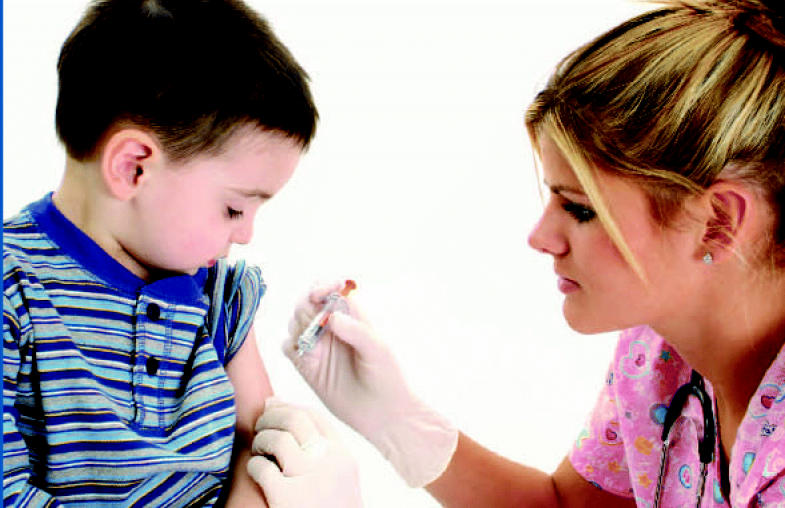# Headliners: Immunity: PCB Exposure Affects Antibody Response in Vaccinated Children

**Published:** 2007-01

**Authors:** Tanya Tillett

Heilmann C, Grandjean P, Weihe P, Nielsen F, Budtz-Jørgensen E. 2006. Reduced antibody responses to vaccinations in children exposed to polychlorinated biphenyls. PLoS Med 3(8):e311.

Although children are known to display varied antibody responses to vaccination, little is known about the causes of these variations. One possibility is that persistent organochlorine pollutants such as polychlorinated biphenyls (PCBs) elicit immunotoxic effects that influence the production of specific antibodies. In this report, NIEHS grantee Philippe Grandjean of the Harvard School of Public Health and his colleagues confirm an association between increased PCB exposure and decreased antibody production in vaccinated children.

The researchers looked at two birth cohorts of generally healthy children (119 children at age 18 months and 129 at age 7 years) living in the Faroe Islands of the North Atlantic, where the traditional diet includes whale blubber that may be contaminated with PCBs. The children were examined after receiving routine childhood vaccinations against tetanus and diphtheria. The researchers determined the children’s exposure to PCBs by measuring PCB concentrations in their mothers’ blood during pregnancy and milk soon after birth, and in the children’s own blood at the time of the study.

The researchers used standard regression analysis techniques to determine the effects of PCB exposure on the production of diphtheria and tetanus antibodies in the children. They found that for each doubling of PCB exposure, diphtheria antibody response decreased by 24% in the 18-month-old children. They also saw a 16% reduction of the tetanus antibody response with each doubling of exposure in the older children. Both prenatal and post-natal exposure seemed to significantly affect antibody concentrations. Although most of the children’s antibody concentrations were well within the range considered necessary to protect against tetanus and diphtheria, the researchers noted diphtheria antibody concentrations below the acceptable limit for long-term protection in 26 of the 7-year-olds two years after receiving a booster vaccination.

Limitations to the study included the small number of children in the two cohorts and the possibility of exposure to other seafood contaminants, such as *p,p*′-DDE. However, the authors say the findings demonstrate evidence of pollutant influences on reduced antibody production after routine childhood vaccination, which could lower a child’s protection against infectious diseases.

## Figures and Tables

**Figure:**